# Parenteral Nutrition Exposure, Nutritional Trajectory, and Clinical Outcomes in Neonates with Surgical Gastrointestinal Conditions: A Retrospective Cohort Study

**DOI:** 10.3390/nu18193251

**Published:** 2026-10-01

**Authors:** Luka Zekovic, Dejan Nikolic, Sanja Sindjic-Antunovic, Biljana Medjo, Dragana Vujovic, Zeljko Grubac, Nina Ristic, Rastko Sava Jeremic, Misela Raus

**Affiliations:** 1 Department of Neonatology, University Children’s Hospital, 11000 Belgrade, Serbia; 2 Faculty of Medicine, University of Belgrade, 11000 Belgrade, Serbia; dejan.nikolic@udk.bg.ac.rs (D.N.);; 3 Department of Physical Medicine and Rehabilitation, University Children’s Hospital, 11000 Belgrade, Serbia; 4 Department of Neonatal Surgery, University Children’s Hospital, 11000 Belgrade, Serbia; 5 Department of Pediatric Intensive Care Unit, University Children’s Hospital, 11000 Belgrade, Serbia; 6 Department for Esophagogastric Disease, Clinic for Digestive Surgery, University Clinical Centre of Serbia, 11000 Belgrade, Serbia; 7 Department of Gastroenterology, Hepatology and GI Endoscopy, University Children’s Hospital, 11000 Belgrade, Serbia

**Keywords:** parenteral nutrition, neonates, surgical gastrointestinal conditions, nutritional trajectory, clinical outcomes, cholestasis, intestinal failure-associated liver disease, catheter-related bloodstream infection, enteral nutrition

## Abstract

**Background/Objectives:** Neonates with surgical gastrointestinal conditions frequently require parenteral nutrition and are exposed to multiple overlapping risks for hepatobiliary, infectious, and nutritional complications. This study aimed to determine whether the timing and initial composition of parenteral nutrition (PN) were independently associated with hepatobiliary dysfunction, treatment-related complications, and nutritional outcomes. **Methods:** This retrospective cohort included 91 neonates hospitalized at the University Children’s Hospital between January 2024 and July 2026 who received parenteral nutrition (PN) for >5 consecutive days. Associations between PN timing, modality, duration, enteral progression, clinical severity, feeding type, and hepatobiliary, infectious, and growth outcomes were assessed using univariable and multivariable analyses. **Results:** Cholestasis occurred in 18 patients (19.8%) and intestinal failure-associated liver disease (IFALD) in 14 (15.4%). The interval from enteral feeding cessation to PN initiation and initial PN modality were not significantly associated with cholestasis or IFALD. Patients with IFALD had longer PN exposure than those without IFALD (21 vs. 12 days; *p* = 0.010). Each additional PN day increased the adjusted odds of catheter-related bloodstream infection by 9.7% (OR 1.097, 95% CI 1.032–1.167; *p* = 0.003). Simultaneous amino-acid and lipid initiation was associated with the highest unadjusted weight-gain velocity and numerically lowest cholestasis and IFALD rates, but no independent protective effect was established. **Conclusions:** Longer PN duration was associated with IFALD and CRBSI, although the IFALD association should be interpreted cautiously because PN duration contributes to its operational definition. Short delays in PN initiation and initial lipid timing were not independently associated with these outcomes. Management should remain individualized, with advancement of enteral nutrition when clinically feasible and minimization of unnecessary PN and central-line exposure.

## 1. Introduction

Neonates admitted with surgical conditions represent a particularly vulnerable population with complex nutritional and metabolic requirements. Although many of these patients undergo operative treatment, a proportion may be managed conservatively after surgical evaluation while still requiring parenteral nutrition (PN) because enteral feeding is temporarily unsafe, insufficient, or clinically unfeasible. Many surgical conditions, sepsis, respiratory instability, and intensive care support may delay the initiation and advancement of enteral nutrition [1,2,3]. In this context, PN remains an essential therapeutic modality, providing energy, amino acids, lipids, electrolytes, vitamins, and trace elements during a period of high metabolic demand and limited nutritional reserves [4].

The pathogenesis of PN-related cholestasis is multifactorial. Prolonged reduction or absence of enteral stimulation may impair cholecystokinin secretion, gallbladder contraction, bile flow, and enterohepatic bile acid circulation. Additional contributors include prematurity, low birth weight, hepatic immaturity, intestinal dysbiosis, bacterial translocation, sepsis, systemic inflammation, overfeeding, lipid-related hepatotoxicity, and micronutrient imbalance [4,5,6]. Among these factors, PN duration remains one of the most consistent predictors of cholestasis. Lauriti et al. demonstrated that PNAC incidence increases with PN duration, while more recent data suggest that the balance between PN and enteral nutrition, expressed through PN duration, enteral nutrition duration, and the PN/EN ratio, may better reflect the risk of PNAC than PN exposure alone [6].

Postoperative nutritional management in neonates at risk of intestinal failure requires a phase-adapted approach. In this context, cautious minimal enteral feeding may be considered after initial clinical stabilization, whereas progressive advancement of enteral nutrition during the recovery phase should be guided by the resolution of the inflammatory response, feeding tolerance, stoma or stool output, and the degree of intestinal adaptation [7]. 

Therefore, in surgical neonates, clinically relevant nutritional outcomes extend beyond the total duration of PN and include the duration of exclusive PN, the period of combined parenteral and enteral nutrition, the timing of enteral feeding initiation, and the time required to achieve full enteral nutrition [8,9,10].

Although previous studies have examined individual components of nutritional management, such as PN duration, timing of enteral feeding, and lipid administration, the relative contribution of the initial PN strategy compared with subsequent PN duration, enteral progression, and clinical severity remains incompletely characterized. In particular, it remains uncertain whether the timing and initial composition of PN are independently associated with hepatobiliary and infectious complications once the subsequent nutritional course and clinical context are considered. A multidimensional assessment integrating these variables into a nutritional trajectory has also been less well characterized.

In the present study, nutritional trajectory was considered the dynamic progression from exclusive parenteral nutrition toward full enteral autonomy. For the purposes of this exploratory study, it was used as a descriptive multidimensional framework rather than as a validated composite score. Its components were selected to represent clinically relevant stages of nutritional recovery and included the timing of PN initiation, the duration of exclusive and total PN, the initiation and progression of enteral feeding, time to full enteral autonomy, and in-hospital weight-gain velocity. These variables were analyzed separately and were not combined into an equally weighted or otherwise weighted index. Accordingly, this study aimed to determine whether the timing of PN initiation following interruption of enteral feeding, the duration and initial modality of PN administration, and the subsequent nutritional trajectory were associated with intestinal failure-associated liver disease (IFALD), cholestasis, catheter-related bloodstream infection, growth, and other clinical outcomes in neonates with surgical gastrointestinal conditions. In this manuscript, nutritional trajectory is therefore proposed as an exploratory conceptual framework rather than a validated prognostic construct or formal composite variable.

## 2. Materials and Methods

### 2.1. Study Design and Setting

This retrospective single-center cohort study included neonates admitted to the University Children’s Hospital in Belgrade, Serbia, between January 2024 and July 2026, who required parenteral nutrition for more than five consecutive days because of congenital or acquired gastrointestinal surgical conditions. The study included both patients who underwent operative treatment and those managed conservatively following surgical evaluation. 

### 2.2. Patient Selection

Patients were eligible for inclusion if they met both of the following criteria: (1) presentation during the neonatal period with a severe congenital or acquired gastrointestinal condition requiring surgical assessment and temporary or prolonged restriction of enteral feeding; and (2) administration of PN for more than five consecutive days. Patients were excluded if relevant clinical, nutritional, or biochemical data were incomplete or if follow-up data required for assessment of the study outcomes were unavailable. 

### 2.3. Definitions

Cholestasis was defined as a serum conjugated bilirubin concentration >34.2 µmol/L (>2 mg/dL), a threshold commonly used in studies evaluating parenteral nutrition-associated cholestasis [11]. Intestinal failure-associated liver disease (IFALD) was defined as cholestasis occurring in the setting of PN administered for more than two weeks, after excluding other identifiable causes of liver disease [12].

Infection at admission was defined as culture-proven infection or clinically suspected infection supported by elevated C-reactive protein and/or procalcitonin concentrations documented at or immediately before transfer from another institution [13].

Catheter-related bloodstream infection (CRBSI) was defined as positive blood cultures obtained from both a central venous catheter and a peripheral vein, in combination with compatible clinical signs and symptoms of infection [14,15,16]. For retrospective outcome ascertainment, CRBSI was assigned only when the predefined microbiological and clinical criteria were documented in the medical record; cases without sufficient documentation to establish these criteria were not classified as CRBSI.

For the purposes of the present exploratory study, nutritional trajectory was defined as the dynamic progression from exclusive PN toward full enteral autonomy and was treated as a descriptive multidimensional framework rather than a validated composite score. It was characterized by the timing of PN initiation, duration of exclusive and total PN, time to initiation and advancement of enteral feeding, time from PN discontinuation to full enteral feeding, and in-hospital weight-gain velocity [17,18]. These components were analyzed individually and were not combined into a weighted index. The components are partly interrelated; for example, total PN duration is closely related to time to enteral autonomy, and exclusive PN duration is related to the timing of enteral feeding initiation. They should therefore not be interpreted as statistically independent dimensions.

### 2.4. Parenteral Nutrition Protocol

Patients were categorized into three parenteral nutrition (PN) modalities according to the composition of the nutritional regimen at initiation and during the course of treatment.

The initial PN modality was not protocol-randomized but was selected by the treating multidisciplinary team, comprising board-certified neonatologists, board-certified pediatric intensivists, and board-certified neonatal surgeons, according to metabolic stability, anticipated duration of PN dependence, biochemical findings, venous access, and the overall clinical condition. Consequently, the three PN groups were not assumed to be clinically exchangeable, and comparisons between regimens were interpreted as observational associations subject to confounding by indication rather than as estimates of comparative treatment effects.

*Type 1 PN* consisted of the initial administration of amino acids—6.5% pediatric crystalline amino acid or 10% crystalline amino acid solution (100 g/L amino acids), together with 10% glucose and 0.9% sodium chloride in an age-adjusted ratio. Amino acids were initiated at a dose of 0.5–1.0 g/kg/day and increased daily by 0.5 g/kg/day up to a maximum dose of 3 g/kg/day, depending on biochemical parameters of renal function. Lipids, administered as 20% mixed-oil intravenous lipid emulsion containing soybean oil, medium-chain triglycerides, olive oil, and fish oil, were subsequently added using the same stepwise advancement regimen, together with vitamin supplementation with parenteral fat-soluble vitamin preparation containing vitamins A, D_2_, E, and K_1_ and parenteral water-soluble multivitamin preparation and pediatric parenteral trace-element solution at a dose of 1 mL/kg body weight were also administered.

*Type 2 PN* consisted exclusively of 6.5% pediatric crystalline amino acid or 10% crystalline amino acid solution (100 g/L amino acids), together with 10% glucose and 0.9% sodium chloride in an age-adjusted ratio. Amino acids were initiated at a dose of 0.5–1.0 g/kg/day and increased daily by 0.5 g/kg/day up to a maximum dose of 3 g/kg/day, according to biochemical parameters of renal function. 

*Type 3 PN* consisted of the simultaneous administration of amino acids, 6.5% pediatric crystalline amino acid solution or 10% crystalline amino acid solution (100 g/L amino acids), and lipids as 20% mixed-oil intravenous lipid emulsion containing soybean oil, medium-chain triglycerides, olive oil, and fish oil, from the initiation of PN, using the same stepwise dose-escalation regimen. Vitamin supplementation with parenteral fat-soluble vitamin preparation containing vitamins A, D_2_, E, and K_1_ and parenteral water-soluble multivitamin preparation and pediatric parenteral trace-element solution at a dose of 1 mL/kg body weight were also administered, together with 10% glucose and 0.9% sodium chloride in an age-adjusted ratio.

### 2.5. Data Collection and Outcome Measures 

The following variables were extracted from the medical records and included in the analysis: *general demographic and perinatal characteristics* (sex, gestational age category, Apgar score, mode of delivery, birth weight, admission body weight, infection at admission, requirement for mechanical ventilation and duration of mechanical ventilation); *underlying neonatal gastrointestinal diagnosis* (including congenital anomalies of the upper and lower gastrointestinal tract, abdominal wall defects, anorectal malformations, congenital diaphragmatic hernia, functional gastrointestinal disorders, biliary tract anomalies, and tumors); *operative characteristics* (requirement for surgical intervention, number of laparotomies, and neonatal intensive care unit length of stay); *enteral nutrition-related variables* (caloric intake before initiation of parenteral nutrition, caloric intake at full enteral feeding, type of milk used for initiation of enteral nutrition, route of enteral feeding initiation, and time to achievement of full enteral nutrition); *parenteral nutrition-related variables* (parenteral nutrition modality, time from enteral feeding cessation to PN initiation, duration of exclusive parenteral nutrition, total duration of parenteral nutrition, duration from PN discontinuation to full enteral feeding, interruption of parenteral nutrition, and venous access type); *clinical complications* (sepsis, catheter-related bloodstream infection, thrombosis, requirement for IgM-enriched immunoglobulin therapy, requirement for blood product transfusion during sepsis, cholestasis, and intestinal failure-associated liver disease); and *clinical outcomes*, including length of hospital stay and body weight at discharge. 

The primary outcomes were the development of cholestasis and IFALD. Secondary outcomes included CRBSI, sepsis, total and exclusive PN duration, progression to full enteral feeding, in-hospital weight-gain velocity, length of hospital stay, and biochemical indicators of hepatobiliary, inflammatory, metabolic, and nutritional status. 

### 2.6. Biochemical Monitoring

All available biochemical measurements obtained during hospitalization were reviewed. For each patient, the clinically most relevant extreme value was extracted according to a prespecified approach: nadir values were used for urea, creatinine, albumin, total protein, calcium, magnesium, phosphorus, and glucose, whereas peak values were used for total and direct bilirubin, ALT, AST, GGT, ALP, CRP, procalcitonin, fibrinogen, total cholesterol, HDL cholesterol, LDL cholesterol, triglycerides, and INR. 

### 2.7. Enteral Nutrition

The type and route of enteral feeding were selected by a multidisciplinary team according to the underlying gastrointestinal condition, gestational and postnatal age, intestinal anatomy and continuity, feeding tolerance, and the overall clinical condition. Enteral feeding included human milk, whole-protein formulas, extensively hydrolyzed or amino-acid-based formulas, and nutrient-enriched formulas. 

### 2.8. Statistical Analysis 

All statistical analyses were performed using *SPSSAU* (*Statistical Product and Service Software Automatically, version 25.0; The SPSSAU Project, online application software*). The distribution of continuous variables was assessed using the Shapiro–Wilk test. Normally distributed continuous variables are presented as the mean and standard deviation (SD), whereas non-normally distributed variables are presented as the median and interquartile range (IQR). Comparisons of normally distributed continuous variables between two independent groups were performed using the independent-samples Student’s *t*-test, while non-normally distributed variables were compared using the two-tailed Mann–Whitney U test. Comparisons of continuous variables across the three parenteral nutrition regimens were performed using the Kruskal–Wallis H test. When an overall statistically significant difference was identified, pairwise post hoc comparisons were performed using Dunn’s test with Bonferroni-adjusted *p* values. Categorical variables are presented as frequencies and percentages and were compared using Pearson’s chi-square test or Fisher’s exact test, as appropriate. For contingency tables larger than 2 × 2 with small expected cell counts, the Fisher–Freeman–Halton exact test was applied. Associations between continuous variables were assessed using Spearman’s rank correlation coefficient (ρ). Effect sizes were reported using *r* for Mann–Whitney U tests, φ for 2 × 2 contingency tables, Cramer’s V for larger contingency tables, and η^2^ for Kruskal–Wallis analyses. 

Time from PN initiation to the first occurrence of cholestasis was analyzed using the Kaplan–Meier method, with comparison among PN modalities performed using the log-rank test. Patients who did not develop cholestasis were censored at PN discontinuation. 

A multivariable linear regression model was used to identify factors independently associated with weight-gain velocity during hospitalization, expressed in g/kg/day. Linear regression results are reported as regression coefficients (β) with corresponding 95% confidence intervals (CIs), whereas logistic regression results are presented as adjusted odds ratios (ORs) with 95% CIs. All statistical tests were two-sided, and a *p* value < 0.05 was considered statistically significant. When multiple comparisons were performed, Bonferroni correction was applied.

## 3. Results

### 3.1. Study Population

The baseline characteristics of the study population are presented in Table 1. A total of 91 neonates requiring parenteral nutrition because of congenital or acquired gastrointestinal disorders were included in the study. Males predominated (61.5%), while 63.7% of patients were born preterm, including 48.3% late preterm, 11.0% very preterm, and 4.4% extremely preterm infants. The most frequent underlying diagnoses were esophageal atresia (17.6%), gastroschisis (11.0%), jejunal and colonic atresia or subocclusion (11.0%). 

### 3.2. Association Between Parenteral Nutrition Regimen and the Development of Cholestasis and IFALD 

IFALD developed in 14 of 91 patients (15.4%), while biochemical cholestasis occurred in 18 (19.8%) (Table 2). Patients with cholestasis had significantly higher maximum GGT, as expected, and AST concentrations than those without cholestasis (360.5 [IQR 208.3–527.5] vs. 187.0 [115.5–272.0] U/L and 108.0 [67.8–215.3] vs. 61.0 [43.0–88.5] U/L, respectively; both *p* < 0.001). Although ALP, ALT, CRP, and procalcitonin were also higher in unadjusted analyses, only AST and GGT remained significant after Bonferroni correction. 

Crude proportions of IFALD and cholestasis did not differ significantly among PN modalities. However, time-to-event analysis demonstrated a borderline significant difference in cumulative cholestasis incidence among the three regimens (log-rank *p* = 0.048) (Table 2). IFALD occurred in 18.2% of neonates receiving amino acids alone, 20.5% of those in whom lipids were introduced subsequently, and 6.7% of those receiving amino acids and lipids from PN initiation (*p* = 0.263). Corresponding cholestasis rates were 31.8%, 20.5%, and 10.0%, respectively (*p* = 0.147). 

Patients who developed IFALD (Table 2) had a significantly longer total duration of PN than those without IFALD (21.0 vs. 12.0 days; *p* = 0.010), while the duration of exclusive PN showed a borderline association (8.0 vs. 5.0 days; *p* = 0.053). Overall, cumulative PN exposure, rather than the specific PN regimen or timing of initiation, showed the strongest association with IFALD development in our study. Because PN exposure for more than two weeks formed part of the operational definition of IFALD, the difference in PN duration between IFALD and non-IFALD groups is partly inherent to the outcome definition and should not be interpreted as an independent risk-factor association. Cholestasis, defined biochemically without incorporating PN duration, was therefore considered the less circular hepatobiliary outcome for etiologic interpretation.

### 3.3. Risk Factors for the Development of CRBSI

CRBSI occurred in 25 neonates and exclusively among patients with a central venous catheter, affecting 55.6% of catheterized patients (Table 3). In univariable analyses, CRBSI was associated with PN modality, PN interruption, thrombosis, sepsis, longer total and exclusive PN exposure, and a longer interval from PN initiation to enteral feeding. Among catheterized neonates, CRBSI was most frequent when lipids were introduced after an initial amino-acid-only period (79.2%), compared with 27.8% when amino acids and lipids were administered from PN initiation (Bonferroni-adjusted *p* = 0.0015).

**Figure 1:**
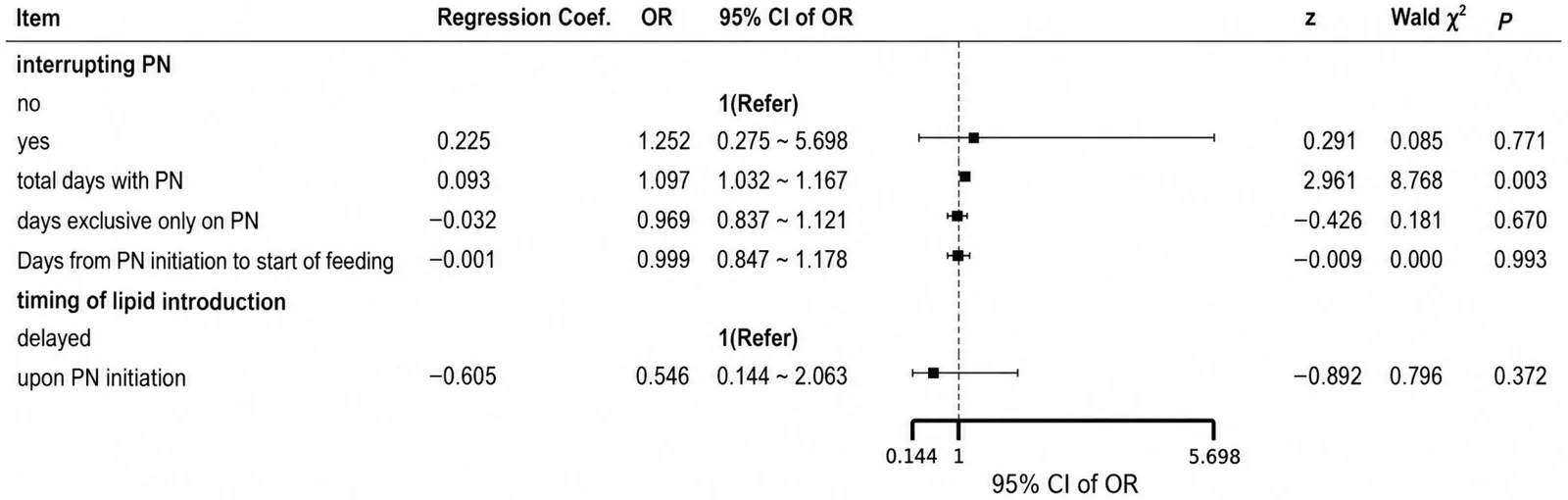
Forest plot of multivariable logistic regression analysis for catheter-related bloodstream infection (CRBSI). The model included PN interruption, total duration of PN, duration of exclusive PN, time from PN initiation to the start of enteral feeding, and timing of lipid introduction. Squares represent adjusted odds ratios (ORs), horizontal lines indicate 95% confidence intervals (CIs), and the vertical dashed line denotes the null value (OR = 1). Total PN duration was the only variable independently associated with CRBSI, with a 9.7% increase in the odds of CRBSI for each additional day of PN (OR 1.097, 95% CI 1.032–1.167; *p* = 0.003). PN interruption, duration of exclusive PN, time to enteral feeding, and timing of lipid introduction were not independently associated with CRBSI.

In the multivariable model (Figure 1), total PN duration was the only variable independently associated with CRBSI, with a 9.7% increase in odds per additional PN day (OR 1.097, 95% CI 1.032–1.167; *p* = 0.003). PN interruption, exclusive PN duration, time to enteral feeding, and initial combined amino-acid and lipid administration were not independently associated with CRBSI. The model showed adequate calibration (Hosmer–Lemeshow *p* = 0.904), high specificity (93.9%), and limited sensitivity (48.0%). CRBSI occurred exclusively among neonates with a central venous catheter, with no CRBSI events among patients with peripheral venous access. Because this produced complete separation, venous access type was not included as a conventional covariate in the multivariable logistic regression model, and no conventional odds ratio for CVC use is reported. Neither PN modality nor PN interruption was significantly associated with cholestasis or IFALD.

### 3.4. Impact of Parenteral Nutrition on Nutritional Trajectory

**Figure 2:**
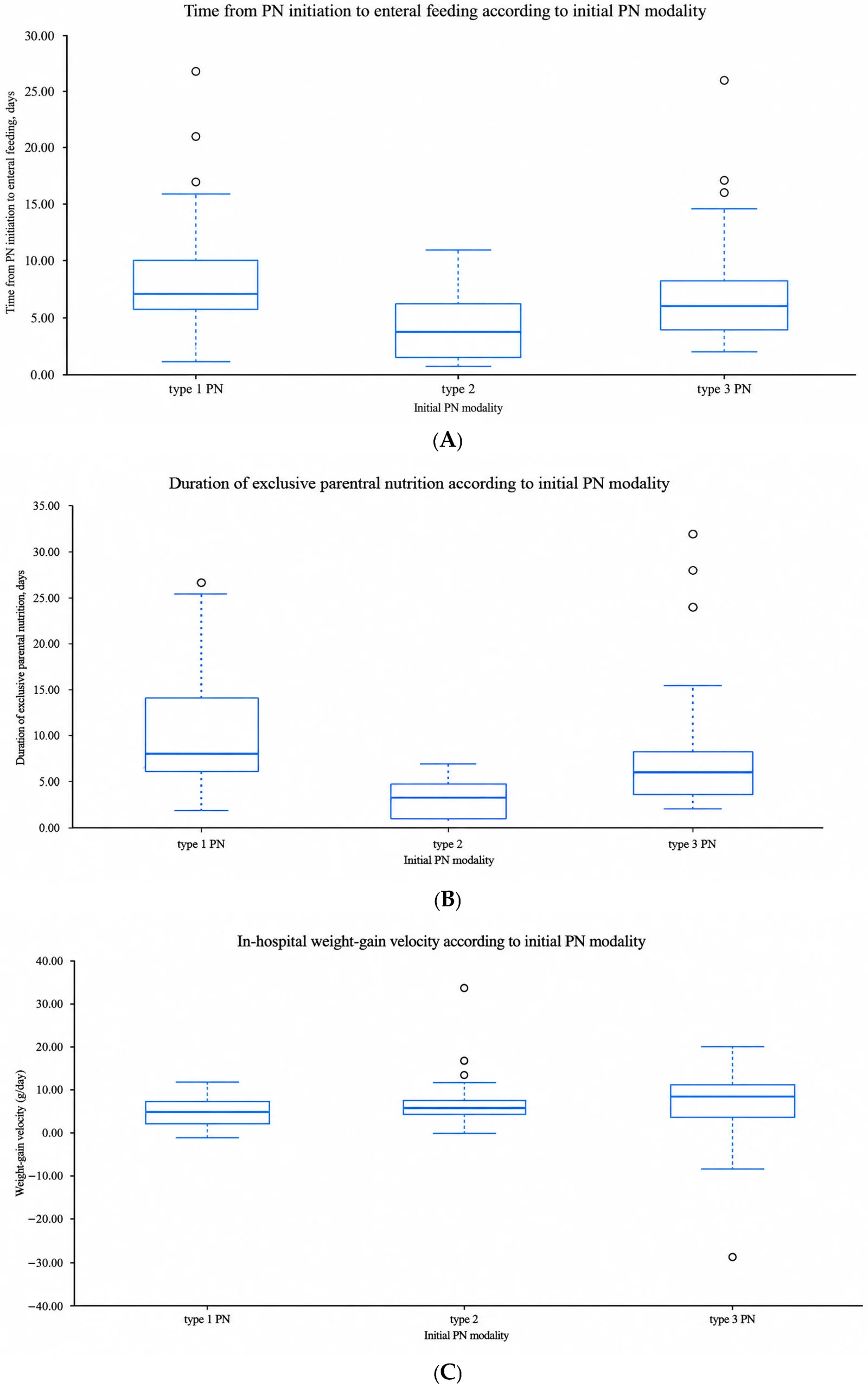
Components of the nutritional trajectory according to initial PN modality. Box plots show (**A**) time from PN initiation to enteral feeding, (**B**) duration of exclusive PN, and (**C**) in-hospital weight-gain velocity. Type 1 PN: amino acids initially with subsequent lipid introduction; Type 2 PN: amino acids without lipid administration; Type 3 PN: simultaneous amino-acid and lipid initiation. Boxes represent the interquartile range and horizontal lines represent medians. Only statistically significant pairwise comparisons are displayed. These comparisons are observational and should not be interpreted as randomized treatment effects.

Initial PN modality was significantly associated with all three assessed components of the nutritional trajectory (Table 4, Figure 2). The interval from PN initiation to the initiation of enteral feeding differed significantly among the three groups (Kruskal–Wallis H = 21.637, *p* < 0.001, η^2^ = 0.223). This interval was significantly shorter among neonates receiving amino acids alone than among those receiving amino acids followed by subsequent lipid introduction (3.5 [IQR 1.8–6.0] vs. 7.0 [6.0–10.0] days; Dunn–Bonferroni adjusted *p* < 0.001) and those receiving amino acids and lipids simultaneously from PN initiation (3.5 [1.8–6.0] vs. 6.0 [4.0–8.3] days; adjusted *p* = 0.011). No significant difference was observed between the delayed-lipid and simultaneous-initiation groups (adjusted *p* = 0.235).

The duration of exclusive PN also differed significantly among the three modalities (H = 24.461, *p* < 0.001, η^2^ = 0.255). Neonates receiving amino acids alone had a significantly shorter duration of exclusive PN than those receiving amino acids followed by lipids (3.5 [IQR 1.0–5.0] vs. 8.0 [6.0–14.0] days; adjusted *p* < 0.001) and those receiving amino acids and lipids from PN initiation (3.5 [1.0–5.0] vs. 6.0 [3.5–8.3] days; adjusted *p* = 0.007). The delayed-lipid and simultaneous-initiation groups did not differ significantly (adjusted *p* = 0.174).

In-hospital weight-gain velocity differed significantly across the three PN modalities (H = 8.823, *p* = 0.012, η^2^ = 0.078). Weight-gain velocity was significantly higher among neonates receiving amino acids and lipids simultaneously from PN initiation than among those in whom lipids were introduced subsequently (8.25 [IQR 3.1–11.1] vs. 4.11 [1.4–6.5] g/kg/day; adjusted *p* = 0.015). The amino-acid-only group did not differ significantly from either the delayed-lipid group (adjusted *p* = 0.130) or the simultaneous-initiation group (adjusted *p* = 1.000). Because PN modality was selected according to clinical status rather than randomized assignment, these between-group differences should be interpreted as descriptive, unadjusted associations and not as comparative effects of biologically equivalent therapeutic strategies.

### 3.5. Association of Initial Versus Delayed Lipid Introduction with Clinical Outcomes

PN modality of initiating lipids was associated with nutritional progression: neonates receiving amino acids alone had the shortest interval to enteral feeding and the shortest duration of exclusive PN, whereas those in whom lipids were introduced subsequently had the longest intervals (both *p* < 0.001). Weight-gain velocity was highest in neonates receiving amino acids and lipids simultaneously from PN initiation (*p* = 0.012). CRBSI and sepsis were most frequent in the delayed-lipid group. Among neonates with a central venous catheter, CRBSI occurred in 79.2% of those with subsequent lipid introduction compared with 27.8% of those receiving simultaneously amino acids and lipids from PN initiation (Bonferroni-adjusted *p* = 0.0015). Nevertheless, initial lipid administration was not independently associated with CRBSI; total PN duration remained the only independent predictor (OR 1.097 per additional PN day, 95% CI 1.032–1.167; *p* = 0.003). 

The delayed-lipid group also showed the least favorable inflammatory and nutritional profile. The initial PN regimen was associated with minimum albumin, calcium, and magnesium concentrations and maximum ALT, CRP, and procalcitonin values. The largest between-group effects were observed for procalcitonin (η^2^ = 0.255), magnesium (η^2^ = 0.213), CRP (η^2^ = 0.207), and albumin (η^2^ = 0.196; all *p* < 0.001). Patients in whom lipids were introduced subsequently had the highest inflammatory marker values and the lowest albumin, calcium, and magnesium concentrations, whereas those receiving amino acids and lipids from PN initiation had the lowest ALT, CRP, and procalcitonin values. These findings represent overall group differences, as pairwise post-hoc comparisons were not performed. No significant differences were found in total or direct bilirubin, AST, GGT, or triglyceride concentrations. 

**Figure 3:**
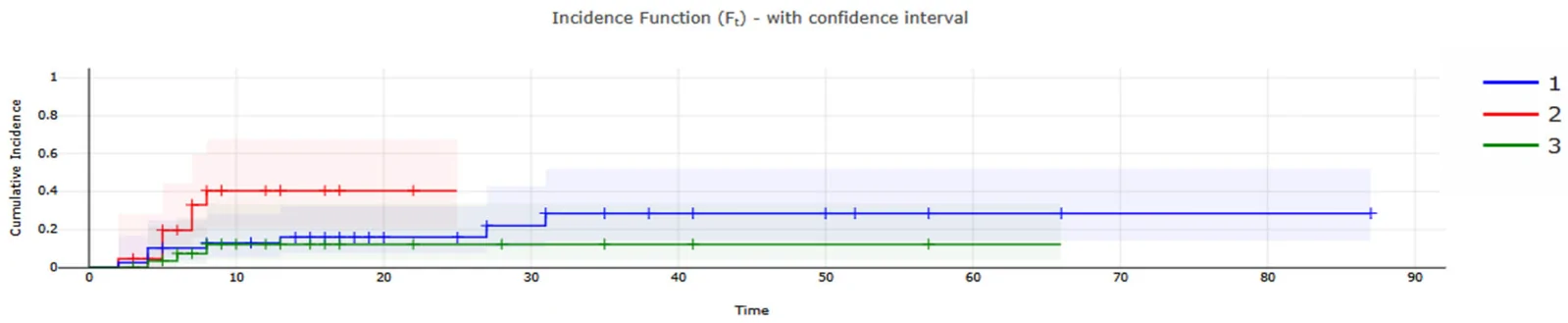
Kaplan–Meier curves for the cumulative incidence of cholestasis according to the initial parenteral nutrition regimen. Group 1 patients receiving amino acids followed by subsequent lipid introduction (Type 1 PN), Group 2 represents patients receiving amino acids alone (Type 2 PN), and Group 3 patients receiving amino acids and lipids from the initiation of parenteral nutrition (Type 3). Shaded areas represent 95% confidence intervals, while tick marks indicate censored observations. The cumulative incidence of cholestasis differed significantly among the three groups (log-rank χ^2^ = 6.087; df = 2; *p* = 0.048). Cholestasis occurred earliest and reached the highest early cumulative incidence in Group 1, whereas Group 3 showed the lowest cumulative incidence throughout follow-up.

Kaplan–Meier analysis (Figure 3) demonstrated a significant difference in the cumulative incidence of cholestasis across the three parenteral nutrition regimens (log-rank χ^2^ = 6.087, df = 2; *p* = 0.048). Cholestasis developed earliest and reached the highest early cumulative incidence in group 1, whereas group 3 showed the lowest cumulative incidence throughout follow-up. Group 2 demonstrated a more gradual accumulation of events over a longer period. Median time to cholestasis was not reached in any group because fewer than 50% of patients developed the event. Given the limited number of events and wide confidence intervals, these findings should be interpreted cautiously. 

## 4. Discussion

### 4.1. Principal Findings and Nutritional Trajectory

In this retrospective cohort, longer PN duration and the broader clinical and nutritional course were associated with adverse outcomes, whereas neither the short interval between enteral feeding cessation and PN initiation nor initial lipid timing showed an independent association with cholestasis or IFALD. Simultaneous initiation of amino acids and lipids was associated with more favorable unadjusted growth outcomes, but no independent protective effect on IFALD or CRBSI was established. Because the PN regimens were selected according to clinical condition, these observations should be interpreted as hypothesis-generating associations rather than causal treatment effects.

Most previous studies investigating IFALD have focused on isolated aspects of parenteral nutrition, such as total PN duration, lipid composition, or individual nutrient doses [6,19]. In contrast, the present study additionally evaluated the temporal progression from PN dependence toward enteral autonomy. This broader framework may provide complementary information regarding nutritional recovery, although it remains exploratory and has not been externally validated.

In the present study, we therefore considered nutritional trajectory as a dynamic process rather than a single nutritional variable. This concept incorporates the timing of PN initiation, duration of exclusive and total PN, progression of enteral feeding, achievement of enteral autonomy, and growth during hospitalization. Together, these variables describe the transition from complete nutritional dependence on parenteral support to physiological enteral nutrition.

From a biological perspective, this approach may better reflect the mechanisms underlying IFALD than isolated nutritional variables. Recovery of enteral feeding restores gastrointestinal motility, stimulates bile flow, promotes enterohepatic circulation, supports intestinal barrier integrity, and reduces dependence on central venous access [19,20]. Consequently, nutritional progression is likely to influence hepatobiliary function, infection risk, and growth simultaneously rather than through a single mechanism.

### 4.2. Incidence of Cholestasis/IFALD and Cumulative PN Exposure

The principal finding was that cumulative PN exposure and the overall clinical and nutritional trajectory appeared to be more strongly associated with adverse outcomes than either the short interval between enteral feeding cessation and PN initiation or the initial timing of lipid administration. Patients who developed IFALD received PN for significantly longer than those without IFALD, whereas neither the timing of PN initiation nor the initial PN modality was significantly associated with cholestasis or IFALD. Furthermore, total PN duration was the only independent predictor of catheter-related bloodstream infection. Simultaneous administration of amino acids and lipids from PN initiation was associated with the highest unadjusted weight-gain velocity and the numerically lowest rates of cholestasis and IFALD; however, these findings were not sufficient to establish an independent protective effect of this regimen.

The incidence of cholestasis observed in our cohort falls within the broad range previously reported in neonates receiving PN. In the systematic review by Lauriti et al., the pooled incidence of PN-associated cholestasis was 28.2%, while IFALD occurred in approximately 49.8% of children with established intestinal failure. Importantly, that review included patients receiving PN for at least 14 days and demonstrated a clear exposure–response relationship, with the incidence of cholestasis increasing from 15.7% in patients receiving PN for up to one month to approximately 60.9% among those exposed for two months or longer [4]. The lower incidence in our cohort may be partly explained by the inclusion of patients receiving PN for more than five days, a median total PN duration of only 14 days, and the inclusion of neonates with transient gastrointestinal dysfunction who did not necessarily have permanent intestinal failure. In comparison, Ardıçlı et al. reported a PNAC incidence of 14.1% in surgical infants, which is broadly comparable to the present findings, although differences in patient selection, PN composition, lipid dose, and duration of exposure limit direct comparison [21].

Patients classified as having IFALD had a longer PN duration than those without IFALD; however, this observation requires particular caution because the operational definition of IFALD used in the present study required PN exposure for more than two weeks. Thus, part of the observed difference in PN duration is structurally related to outcome classification and cannot be interpreted as an independent risk-factor association. By contrast, cholestasis was defined biochemically without incorporating PN duration and therefore provides a less circular outcome for assessing hepatobiliary associations. The duration of exclusive PN showed a borderline association with IFALD, but this finding should likewise be interpreted in the context of the underlying disease course and delayed enteral autonomy.

Taken together, previous studies suggest that no single modification of PN composition is sufficient to prevent IFALD. Instead, IFALD appears to result from the interaction of prolonged PN exposure, intestinal dysfunction, systemic inflammation, reduced enteral stimulation, and disease severity [22,23]. Our findings support this multifactorial concept by demonstrating that cumulative PN exposure and nutritional progression were more strongly associated with adverse outcomes than the initial timing of lipid administration. Accordingly, prolonged PN should be interpreted both as a biologically plausible contributor to hepatobiliary injury and as a marker of more severe gastrointestinal dysfunction, delayed enteral autonomy, and greater overall clinical complexity. 

### 4.3. Timing of PN Initiation

In contrast to cumulative PN exposure, the time from enteral feeding cessation to PN initiation was not associated with cholestasis, IFALD, or CRBSI. This finding suggests that, within the relatively narrow initiation interval observed in our cohort, a delay of several days was less relevant than the subsequent duration of PN dependence. This result should not be interpreted as evidence that PN can be universally initiated early or withheld without consequence. In the PEPaNIC randomized trial, withholding PN during the first week of pediatric intensive care was associated with fewer new infections and shorter ICU dependency than early PN [24]. However, that trial enrolled a broad pediatric intensive care population and excluded important groups such as premature neonates and children with short bowel syndrome or conditions requiring immediate PN. Its findings therefore cannot be directly extrapolated to neonates with congenital or acquired gastrointestinal surgical disorders, who have limited nutrient reserves and may be unable to establish meaningful enteral intake. In the present population, PN initiation should consequently remain individualized according to metabolic stability, pre-existing nutritional intake, expected duration of enteral restriction, gestational age, and disease severity rather than being delayed solely because of concern for cholestasis.

### 4.4. Initial Lipid Strategy and Confounding by Indication

The absence of a statistically significant association between the initial PN modality and either cholestasis or IFALD is clinically relevant. Although both outcomes were numerically least frequent among neonates receiving amino acids and 20% mixed-oil intravenous lipid emulsion containing soybean oil, medium-chain triglycerides, olive oil, and fish oil simultaneously from PN initiation, the differences did not reach statistical significance. Importantly, the present study evaluated the timing of lipid introduction rather than comparing different intravenous lipid emulsions, because 20% mixed-oil intravenous lipid emulsion containing soybean oil, medium-chain triglycerides, olive oil, and fish oil, was used whenever parenteral lipids were administered. Accordingly, these findings cannot be interpreted as evidence of the superiority of one lipid emulsion over another.

Initial PN modality was, however, significantly associated with several components of the subsequent nutritional trajectory. Neonates receiving amino acids alone had a significantly shorter interval from PN initiation to enteral feeding than those receiving amino acids followed by delayed lipid introduction and those receiving amino acids and lipids simultaneously from PN initiation. They also had a significantly shorter duration of exclusive PN than both lipid-containing groups. 

Because the initial PN regimen was not randomized but was selected according to anticipated PN dependence, metabolic stability, venous access, biochemical findings, and overall clinical condition, amino-acid-only PN may have been preferentially administered to neonates expected to require shorter nutritional support and to resume enteral feeding earlier. Comparisons among PN regimens should therefore be interpreted as observational associations rather than treatment effects. The observed differences may reflect treatment selection and confounding by indication, and residual confounding cannot be excluded despite multivariable adjustment. Accordingly, the three PN modalities cannot be interpreted as equivalent therapeutic strategies, and the pattern of longer exclusive PN, delayed enteral feeding, greater inflammatory burden, and longer hospitalization in the delayed-lipid group is compatible with preferential selection of that regimen in more severely ill or clinically complex patients.

The delayed-lipid group had the least favorable overall unadjusted clinical, inflammatory, and nutritional profile. These patients had the highest frequencies of sepsis and CRBSI, the highest CRP and procalcitonin concentrations, and the lowest albumin, calcium, and magnesium concentrations during hospitalization. Although their median interval to enteral feeding and duration of exclusive PN were the longest among the three groups, post hoc analyses showed that these differences were significant only in comparison with the amino-acid-only group and not in comparison with simultaneous amino-acid and lipid initiation. Nevertheless, the simultaneous-initiation group did not differ significantly from the amino-acid-only group, and the association with weight gain remained unadjusted and was influenced by substantial interindividual variability and extreme observations. Differences in fluid balance, disease severity, length of hospitalization, and timing of weight measurements may also have contributed. This finding should therefore be regarded as hypothesis-generating rather than as evidence of an independent growth-promoting effect of early lipid administration.

Furthermore, the initial lipid strategy was not independently associated with CRBSI, whereas total PN duration remained the only independent predictor in the multivariable model. This further supports the likelihood of confounding by indication. Clinicians may have delayed lipid introduction in neonates who were more unstable, septic, metabolically compromised, or expected to require more complex and prolonged nutritional management [25,26]. The relevance of underlying clinical severity in neonatal surgical patients is supported by previous findings from our center, where preoperative mechanical ventilation and postoperative sepsis were independently associated with late postoperative complications in neonates with esophageal atresia [27]. Consequently, the delayed-lipid regimen may represent a marker of greater baseline or evolving clinical severity rather than the cause of the adverse biochemical, infectious, and nutritional profile. Taken together, these results suggest that the initial PN modality was closely related to the subsequent nutritional trajectory, but that the observed differences reflected both nutritional exposure and the clinical context in which each regimen was selected.

### 4.5. Nutritional Progression, Growth, and Enteral Feeding

Conversely, the simultaneous introduction of amino acids and lipids was associated with the highest median weight-gain velocity. This finding is physiologically plausible because early provision of both protein and a calorie-dense lipid source may facilitate a more balanced non-protein energy-to-protein ratio, reduce endogenous protein catabolism, and support anabolism during recovery [28,29,30]. However, this association was unadjusted, and total PN duration was not independently associated with weight-gain velocity in the multivariable model. Furthermore, the expanded regression model was not statistically significant and contained influential observations. The apparent growth advantage should therefore be interpreted as hypothesis-generating rather than as evidence that simultaneous lipid introduction independently improves growth.

Human milk was associated with a higher weight-gain velocity in the parsimonious regression model, with an estimated increase of 7.17 g/kg/day compared with the reference formula category. Cimadamore et al. summarized retrospective evidence indicating a lower prevalence of PN-associated liver disease and lower peak conjugated bilirubin concentrations in infants fed exclusively with human milk compared with formula-fed infants [19]. However, the available evidence remains limited, and several reported associations have not persisted after adjustment for disease severity and intestinal anatomy. In our cohort, only 12 patients received human milk, and the expanded regression model was unstable. Therefore, although the result supports prioritizing maternal or donor human milk when clinically and anatomically feasible, it requires confirmation in larger cohorts with more detailed adjustment for diagnosis, gestational age, bowel anatomy, enteral volume, and feeding tolerance.

### 4.6. CRBSI, Central Venous Access, and Inflammation

The association between PN duration and CRBSI was among the more robust findings of the study. CRBSI occurred exclusively in neonates with central venous catheters, and longer PN duration was independently associated with higher odds of CRBSI in the prespecified multivariable model. Because no CRBSI events occurred among patients with peripheral venous access, access type produced complete separation and was not entered as a conventional predictor in that logistic regression model. Longer PN duration generally implies greater central-line exposure, catheter manipulation, and opportunity for bloodstream infection [31]; however, central-line days were not available as a separate exposure variable. Reverse causation must also be considered, as CRBSI and sepsis can interrupt feeding, worsen clinical status, and prolong both PN and central venous access [32].

The exclusive occurrence of CRBSI among patients with central venous access emphasizes the clinical importance of minimizing unnecessary catheter exposure, but it does not permit a stable conventional odds-ratio estimate for CVC use in this dataset. Central-line infections are particularly relevant in the context of hepatobiliary dysfunction because inflammatory signaling during bacteremia and sepsis may impair hepatocellular bile-acid transport and exacerbate cholestasis [33]. Contemporary neonatal infection-prevention literature likewise emphasizes standardized insertion and maintenance practices and prompt removal of central lines when they are no longer required [34].

### 4.7. Biochemical and Metabolic Findings

The laboratory findings further support the coexistence of cholestatic and hepatocellular injury. After correction for multiple comparisons, maximum GGT and AST concentrations remained significantly higher among neonates with cholestasis. Elevated GGT is compatible with impaired bile flow and biliary epithelial injury, whereas AST is less specific and may also reflect hepatocellular, muscular, ischemic, or systemic inflammatory injury [35,36]. Although ALT, ALP, CRP, and procalcitonin were higher in the initial analyses, their loss of statistical significance after Bonferroni correction indicates that these abnormalities should not be interpreted as specific markers of PN-related liver disease. Direct bilirubin should remain the principal biochemical measure for defining and monitoring cholestasis, while GGT and aminotransferase trends may provide complementary information regarding the pattern and evolution of liver injury [21]. Future studies may examine whether composite indices such as the AST-to-Platelet Ratio Index (APRI) provide additional information regarding liver injury, although their neonatal interpretation is limited by the sensitivity of platelet counts to sepsis and other non-hepatic conditions [37,38].

Our study demonstrates that longer periods of PN without enteral feeding and PN interruptions were also associated with lower minimum magnesium, phosphorus, calcium, albumin, and total protein concentrations. These findings indicate a vulnerable metabolic and nutritional state but should not be attributed to PN composition alone [22,26]. Nevertheless, these associations reinforce the need for serial monitoring and individualized adjustment of electrolytes, minerals, protein, vitamins, and trace elements, particularly during the transition from the acute catabolic phase to recovery and renewed anabolism [33].

### 4.8. Clinical Implications

To date, no commercially available intravenous lipid emulsion (ILE) fully reproduces the tightly regulated intrauterine supply [39]. Our study evaluated the timing of lipid introduction rather than the composition of intravenous lipid emulsions. Although simultaneous initiation of amino acids and lipids was associated with numerically lower rates of cholestasis and IFALD, these associations were not statistically significant after adjustment for confounding variables. These findings suggest that, within the context of a standardized 20% mixed-oil intravenous lipid emulsion containing soybean oil, medium-chain triglycerides, olive oil, and fish oil-based protocol, cumulative PN exposure and the underlying clinical course may be more important determinants of hepatobiliary outcomes than the precise timing of lipid initiation.

Current neonatal nutritional guidance supports early amino-acid provision to cover basal protein requirements, with subsequent phase-adapted advancement of macronutrient intake according to metabolic stability and tolerance [26,33,40]. Appropriate energy delivery remains an essential component of neonatal PN. Excessive glucose administration may contribute to hepatic steatosis [41], whereas inadequate energy intake may impair growth and delay recovery [33]. Although cumulative glucose exposure was not evaluated in the present study, individualized metabolic monitoring remains important when optimizing PN in critically ill neonates.

### 4.9. Strengths and Limitations

The study population was clinically heterogeneous, including neonates with a broad spectrum of congenital and acquired gastrointestinal disorders that differ substantially in intestinal anatomy, expected duration of parenteral nutrition, disease severity, and risk of hepatobiliary complications. Although this heterogeneity reflects real-world clinical practice, it may have introduced substantial residual confounding and reduced our ability to detect diagnosis-specific associations. Because PN dependence is strongly determined by underlying gastrointestinal anatomy, disease severity, postoperative course, and the capacity to achieve enteral autonomy, total PN duration should be interpreted both as a biologically plausible exposure and as a marker of underlying clinical complexity.

The initial parenteral nutrition regimen was selected according to clinical judgment rather than random allocation. Consequently, delayed lipid introduction may have been preferentially used in clinically unstable or metabolically compromised neonates, introducing confounding by indication. The observed associations between PN modality and adverse outcomes may therefore reflect baseline or evolving disease severity rather than the nutritional strategy itself. Accordingly, comparisons among PN modalities should not be interpreted as evidence of comparative efficacy or causality. Residual confounding may additionally arise from disease severity, indication for CVC placement, anticipated duration of venous access, and clinician decision-making that influenced both PN modality and the pace of enteral progression.

Despite including 91 neonates, only 18 patients developed cholestasis and 14 developed IFALD. The limited number of outcome events restricted the number of covariates that could be included in multivariable analyses and may have reduced statistical power to detect independent associations. Although diagnosis-specific subgroup analyses would be clinically informative, further subdivision of the cohort would have produced very small groups with unstable estimates; therefore, such analyses were not considered sufficiently robust for the present dataset.

Consequently, the regression models should be interpreted cautiously, as the relatively small number of outcome events increases the possibility of model overfitting and unstable regression estimates despite careful variable selection. Larger multicenter cohorts will be required for adequately powered diagnosis-specific analyses and more comprehensive adjustment for disease severity. CRBSI occurred only among neonates with central venous access, producing complete separation between access type and CRBSI status. Conventional logistic regression therefore could not provide a reliable estimate of the effect of CVC use on CRBSI, and access type was not included as a conventional covariate in the CRBSI model. In addition, retrospective CRBSI ascertainment depended on the availability and completeness of paired microbiological and clinical documentation.

The study did not evaluate cumulative macronutrient exposure, glucose infusion rates, cumulative lipid dose, cumulative amino acid dose, daily parenteral energy delivery, or cumulative energy intake. The absence of these quantitative exposure data prevents assessment of dose–response relationships between individual macronutrients and hepatobiliary outcomes and limits mechanistic interpretation. Accordingly, the present study evaluates the timing and duration of nutritional strategies rather than the metabolic effects of cumulative nutrient administration. These variables should be incorporated into future prospective investigations.

Similarly, detailed surgical variables such as residual bowel length, extent of intestinal resection, presence of an ileocecal valve, stoma characteristics, and precise measures of intestinal continuity were not available for analysis. These factors are known determinants of intestinal failure and may substantially influence both PN duration and IFALD risk [12].

Although we introduced nutritional trajectory as a multidimensional framework for nutritional recovery, this construct has not yet been externally validated. It should therefore be regarded as exploratory rather than as an established nutritional outcome metric. Its individual components were analyzed separately rather than combined into a weighted score. Prospective studies are needed to determine whether this framework provides additional prognostic value compared with conventional measures such as total PN duration or time to full enteral feeding.

Despite these limitations, the study reflects routine clinical practice in a tertiary neonatal surgical center and provides comprehensive data on nutritional progression, hepatobiliary complications, and infectious outcomes. These findings may serve as a foundation for future multicenter prospective studies using standardized nutritional protocols and more detailed assessment of disease severity.

### 4.10. Future Perspectives

Future multicenter prospective studies should use standardized PN protocols and prospectively collect cumulative lipid, amino-acid, glucose, and total energy exposure; detailed gastrointestinal anatomy and surgical variables; validated measures of disease severity; central-line days; and longitudinal enteral-feeding progression. Such studies should also determine whether a formally defined and externally validated nutritional trajectory provides incremental prognostic information beyond conventional variables such as total PN duration, time to enteral autonomy, and underlying diagnosis. This approach may help distinguish effects related to nutritional exposure from those driven primarily by disease severity and clinical decision-making [34,42].

## 5. Conclusions

In this retrospective cohort of neonates with surgical gastrointestinal conditions, longer duration of parenteral nutrition was associated with IFALD and CRBSI, although the association with IFALD should be interpreted cautiously because PN duration forms part of its operational definition. The short interval between enteral feeding interruption and PN initiation was not significantly associated with cholestasis, IFALD, or CRBSI. Initial lipid timing was not independently associated with IFALD or CRBSI. Simultaneous amino-acid and lipid initiation was associated with higher unadjusted weight-gain velocity, but this finding should be regarded as hypothesis-generating. These observations support individualized nutritional management, advancement of enteral nutrition when clinically feasible, and minimization of unnecessary PN and central venous catheter exposure.

The findings should not be interpreted as evidence of causality or comparative efficacy between PN regimens, because treatment allocation was non-randomized and influenced by clinical condition.

## Figures and Tables

**Table 1:** Descriptive statistics of the study population.

Variables		Values
No. of patients		91
Female sex, *N* (%)		35 (38.5%)
Male sex, *N* (%)		56 (61.5%)
Maturity, *N* (%)		
	term	33 (36.3%)
	early preterm	44 (48.3%)
	very preterm	10 (11.0%)
	extreme preterm	4 (4.4%)
Apgar score, median (IQR)		8 (7, 9)
Delivery, *N* (%)	vaginal	29 (31.9%)
	section	62 (68.1%)
Birth weight, grams, median (IQR)		2575.0 (1960.0, 3320.0)
Admission body weight, median (IQR)		2600.0 (2010.0, 3320.0)
Infection at admission, *N* (%)	Yes	14 (15.4%)
	No	77 (84.6%)
Mechanical ventilation (MV), *N* (%)		48 (72.4%)
	Duration of MV (hours), median (IQR)	84.5 (30.0, 101.0)
**Diagnoses occurring in the neonatal period**		
Diseases of the upper GUT, *N* (%)		
	esophageal atresia *	16 (17.6%)
	duodenal atresia	5 (5.5%)
	gastric perforation	1 (1.1%)
Diseases of the lower GUT, *N* (%)		
	jejunal and colonic atresia/subocclusion	10 (11.0%)
	NEC	7 (7.7%)
	perforated	4 (4.4%)
	meconium ileus	8 (8.8%)
	malrotation/volvulus	7 (7.7%)
Abdominal wall defect, *N* (%)	gastroschisis	10 (11.0%)
	umbilical cord hernia	1 (1.1%)
Anorectal anomalies, *N* (%)	anal atresia	2 (2.2%)
	cloaca	2 (2.2%)
Congenital diaphragmatic hernia, *N* (%)		7 (7.7%)
Functional gastrointestinal disorders, *N* (%)	hypomotility	8 (8.8%)
	megacolon	4 (4.4%)
Tumors, *N* (%)	sacrococcygeal teratoma	1 (1.1%)
	abdominal teratoma	1 (1.1%)
Biliary tract anomaly, *N* (%)	choledochal cyst	1 (1.1%)
**Operation information**		
No. of patients undergoing surgery, *N* (%)		84 (92.3%)
NICU length of stay (days), median (IQR)		4 (0, 9)
**EN information**		
Caloric intake at full enteral feeding (kcal/kg/day), median (IQR)		111.3 (92.4, 129.5)
Human milk		12 (13.2%)
Type of formula milk, *N* (%)		
	(1) Whole protein	14 (15.4%)
	(2) AAF and eHF	47 (51.6%)
	(3) Nutrient-enriched	18 (19.8%)
EN feed route initiation, *N* (%)		
	Oral feeding	50 (55.0%)
	Nasogastric tube	41 (45.0%)
		
**PN information**		
Caloric intake before PN initiation (kcal/kg/day), median (IQR)		33.0 (26.0, 40.0)
Modality of PN, *N* (%)		
	Start with amino acids, later added lipids	39 (42.9%)
	Amino acids only	22 (24.2%)
	Amino acids and lipids from PN initiation	30 (32.9%)
Timeline of PN, median (IQR)		
	Days from enteral feeding cessation to PN initiation (days), median (IQR)	2 (1, 3)
	Days on exclusive parenteral nutrition, median (IQR)	6 (4, 9)
	Total duration of parenteral supplementation (days), median (IQR)	14 (8, 24)
	Days from PN discontinuation to full enteral feeding, median (IQR)	3 (2, 5)
	Interruption of parenteral nutrition, *N* (%)	21 (23.1%)
Venous access, *N* (%)		
	CVC	45 (49.5%)
	PVC	46 (50.5%)
**Complications**		
	Sepsis, *N* (%)	38 (41.8%)
	Requirement for enriched immunoglobulin therapy, *N* (%)	23 (60.5%)
	Need for blood product transfusion during sepsis, *N* (%)	24 (63.2%)
	Thrombosis, *N* (%)	12 (26.7%) **
	IFALD, *N* (%)	14 (15.4%)
	CRBSIs, *N* (%)	25 (27.5%)
**Outcomes**		
	LOS, median (IQR)	23 (15, 42)
	Weight-gain velocity (g/kg/day), median (IQR)	5.0 (2.36, 8.39)

AAF, Amino acid-based formulas; CRBSIs, Catheter-related bloodstream infections; CVC, Central vein catheter; eHF, Extensively hydrolyzed protein formula; EA, esophageal atresia; EN, enteral nutrition; GUT, gastrointestinal tract; IFALD, Intestinal Failure-Associated Liver Disease; MV, Mechanical ventilation; NEC, necrotizing enterocolitis; NICU, neonatal intensive care; LOS, Length of Stay; PN, Parenteral Nutrition; PVC, Peripheral vein catheter; TEF, tracheoesophageal fistula. * EA with and without TEF. ** percentage of patients with CVC.

**Table 2:** Distribution of tested variables with regard to the presence of Intestinal Failure Liver Disease.

		IFALD (%)		*p* Value
		Non-IFALD (*N* = 77)	IFALD (*N* = 14)	
**General**				
Sex, *N* (%)	Female	33 (94.3%)	2 (5.7%)	0.043 *
	Male	44 (78.6%)	12 (21.4%)	
Maturity, *N* (%)	Term	31 (93.9%)	2 (6.1%)	0.209
	Early preterm	34 (77.3%)	10 (22.7%)	
	Very preterm	9 (90.0%)	1 (10.0%)	
	Extreme preterm	3 (75.0%)	1 (25.0%)	
Apgar score, median (Q1,Q3)		8 (7.0, 9.0)	7 (6.0, 9.0)	0.277
Admission body weight, median (Q1,Q3)		2680.0 (2010.0, 3370.0)	2200.0 (1685.0, 2492.5)	0.073
**Intensive care**				
MV (duration in hours), median (Q1,Q3)		12.0 (0.0, 88.5)	15.0 (0.0, 87.5)	0.907
NICU days, median (Q1,Q3)		5.0 (0.0, 8.5)	2.0 (0.0, 20.5)	0.889
**EN information**				
Caloric intake at full enteral feeding, median (Q1,Q3)		112.7 (95.5, 130.5)	102.6 (84.8, 121.1)	0.257
Milk type, *N* (%)	Human	10 (83.3%)	2 (16.7%)	0.254
	Whole protein	14 (100.0%)	0 (0.0%)	
	AAF and eHF	37 (78.7%)	10 (21.3%)	
	Nutrient-enriched	16 (88.9%)	2 (11.1%)	
Enteral feeding initiation	Oral	40 (80.0%)	10 (20.0%)	0.178
	Nasogastric (NG) tube	37 (90.2%)	4 (9.8%)	
**PN Information**				
Caloric intake before PN, median (Q1,Q3)		32.0 (25.0, 39.0)	39.5 (31.0, 42.0)	0.015 *
Modality of PN, *N* (%)	Type1	31 (79.5%)	8 (20.5%)	0.263
	Type2	18 (81.8%)	4 (18.2%)	
	Type3	28 (93.3%)	2 (6.7%)	
Timeline of PN, median (Q1,Q3)	Days from enteral feeding cessation to PN initiation (days)	2.0 (1.0, 3.0)	1.5 (1.0, 2.3)	0.657
	Days from initiating of PN to start feeding	6.0 (4.0, 8.0)	7.5 (5.5, 14.8)	0.084
	Days from PN excluding to full enteral feeding	3.0 (2.0, 5.0)	2.5 (1.8, 5.0)	0.396
Interruption of PN, *N* (%)	No	61 (87.1%)	9 (12.9%)	0.222
	Yes	16 (76.2%)	5 (23.8%)	
Venous access, *N* (%)	PVC	41 (89.1%)	5 (10.9%)	0.227
	CVC	36 (80.0%)	9 (20.0%)	
**Complications and Outcomes**				
CRBSI, *N* (%)	No	57 (86.4%)	9 (13.6%)	0.453
	Yes	20 (80.0%)	5 (20.0%)	
NICU days, median (Q1,Q3)		5.0 (0.0, 8.5)	2.0 (0.0, 20.5)	0.889
Weight-gain velocity (g/kg/day), median (Q1,Q3)		5.003 (2.3, 8.3)	4.685 (2.5, 8.8)	0.869
LOS, median (Q1,Q3)		22.0 (13.5, 41.0)	30.5 (22.3, 52.3)	0.040 *

* *p* < 0.05, AAF, Aminoacid-based formulas; CRBSI, Catheter-related bloodstream infections; CVC, Central vein catheter; eHF, Extensively hydrolyzed protein formula; EN, enteral nutrition; IFALD, Intestinal Failure-Associated Liver Disease; MV, Mechanical ventilation; NICU, neonatal intensive care; LOS, Length of Stay; PN, Parenteral Nutrition; PVC, Peripheral vein catheter.

**Table 3:** Distribution of tested variables with regard to the presence of Catheter-related bloodstream infections.

		CRBSI (%)		*p* Values
		Non-CRBSI (*N* = 66)	CRBSI (*N* = 25)	
**General**				
Sex, *N* (%)	Female	24 (68.6%)	11 (31.4%)	0.504
	Male	42 (75.0%)	14 (25.0%)	
Maturity, *N* (%)	Term	23 (69.7%)	10 (30.3%)	0.094
	Early preterm	29 (65.9%)	15 (34.1%)	
	Very preterm	10 (100.0%)	0 (0.0%)	
	Extreme preterm	4 (100.0%)	0 (0.0%)	
Apgar score, median (IQR)		8.0 (6.0, 9.0)	8.0 (7.0, 9.0)	0.595
Admission body weight, median (Q1,Q3)		2355.000 (1945.0, 3102.5)	2850.000 (2400.0, 3410.0)	0.028 *
**Intensive care**				
MV (duration in hours), median (Q1,Q3)		0.0 (0.0, 84.3)	19.0 (0.0, 90.5)	0.342
NICU days, median (Q1,Q3)		4.0 (0.0, 8.0)	5.0 (0.0, 11.0)	0.314
**EN information**				
Caloric intake at full enteral feeding, median (Q1,Q3)		118.1 (97.5, 131.1)	102.7 (89.2, 124.2)	0.164
Milk type, *N* (%)	Human	9 (75.0%)	3 (25.0%)	0.251
	Whole protein	12 (85.7%)	2 (14.3%)	
	AAF and eHF	30 (63.8%)	17 (36.2%)	
	Nutrient-enriched	15 (83.3%)	3 (16.7%)	
Enteral feeding initiation, *N* (%)	Oral	40 (80.0%)	10 (20.0%)	0.078
	Nasogastric (NG) tube	26 (63.4%)	15 (36.6%)	
**PN Information**				
Caloric intake before PN				
Modality of PN, *N* (%)	Type1	20 (51.3%)	19 (48.7%)	<0.01 **
	Type2	21 (95.4%)	1 (4.5%)	
	Type3	25 (83.3%)	5 (16.7%)	
Timeline of PN, median (Q1,Q3)	Days from enteral feeding cessation to PN initiation (days)	1.0 (1.0, 3.0)	2.0 (1.0, 3.0)	0.111
	Days from initiating of PN to start feeding	6.0 (3.8, 8.0)	7.0 (5.5, 10.0)	0.038 *
	Days from PN excluding to full enteral feeding	3.0 (2.0, 5.0)	3.0 (2.0, 5.5)	0.591
Interruption of PN, *N* (%)	No	57 (81.4%)	13 (18.6%)	0.001 **
	Yes	9 (42.9%)	12 (57.1%)	
Venous access, *N* (%)	PVC	46 (100.0%)	0 (0.0%)	<0.01 **
	CVC	20 (44.4%)	25 (55.6%)	
**Complications and Outcomes**				
IFALD, *N* (%)	No	57 (74.0%)	20 (26.0%)	0.453
	Yes	9 (64.3%)	5 (35.7%)	
NICU days, *N* and median (Q1,Q3)		4.0 (0.0, 8.0)	5.0 (0.0, 11.0)	0.314
Weight-gain velocity (g/kg/day, median (Q1,Q3)		5.79 (2.9, 9.1)	3.60 (1.4, 6.5)	0.030 *
LOS, median (Q1,Q3)		21.5 (12.8, 33.3)	44.0 (24.5, 54.0)	<0.01 **

* *p* < 0.05 ** *p* < 0.01, AAF, Aminoacid-based formulas; CRBSIs, Catheter-related bloodstream infections; CVC, Central vein catheter; eHF, Extensively hydrolyzed protein formula; EN, enteral nutrition; IFALD, Intestinal Failure-Associated Liver Disease; MV, Mechanical ventilation; NEC, necrotizing enterocolitis; NICU, neonatal intensive care; LOS, Length of Stay; PN, Parenteral Nutrition; PVC, Peripheral vein catheter.

**Table 4:** Distribution of tested variables regarding different Parenteral Nutrition Modalities.

		PN Modality			*p* Value
		Type 1 (*n* = 39)	Type 2 (*n* = 22)	Type 3 (*n* = 30)	
**General**					
Sex, *N* (%)	Female	16 (45.7%)	10 (28.6%)	9 (25.7%)	0.479
	Male	23 (41.1%)	12 (21.4%)	21 (37.5%)	
Maturity, *N* (%)	Term	16 (48.5%)	4 (12.1%)	13 (39.4%)	0.451
	Early preterm	19 (43.2%)	13 (29.5%)	12 (27.3%)	
	Very preterm	3 (30.0%)	3 (30.0%)	4 (40.0%)	
	Extreme preterm	1 (25.0%)	2 (50.0%)	1 (25.0%)	
Apgar score, median (IQR)		8.0 (2.0)	8.0 (2.8)	8.0 (2.0)	
Admission body weight, median (Q1,Q3)		2690.0 (2150.0, 3370.0)	2285.0 (1952.5, 2755.0)	2597.5 (1690.0, 3347.5)	0.283
**Intensive care**					
MV (duration in hours), median (IQR)		20.0 (96.0)	0.0 (0.0)	27.5 (90.8)	0.001 **
NICU days, median (IQR)		5.0 (9.5)	6.0 (7.3)		<0.001 **
**EN information**					
Caloric intake at full enteral feeding, median (Q1,Q3)		104.3 (91.9, 127.0)	119.4 (88.2, 140.2)	111.9 (97.7, 129.3)	0.739
Milk type, *N* (%)	Human	6 (50.0%)	5 (41.7%)	1 (8.3%)	0.262
	Whole protein	3 (21.4%)	4 (28.6%)	7 (50.0%)	
	AAF and eHF	22 (46.8%)	10 (21.3%)	15 (31.9%)	
	Nutrient-enriched	8 (44.4%)	3 (16.7%)	7 (38.9%)	
Enteral feeding initiation, median (IQR)	Oral	17 (34.0%)	18 (36.0%)	15 (30.0%)	0.013 *
	Nasogastric (NG) tube	22 (53.7%)	4 (9.8%)	15 (36.6%)	
**PN Information**					
Caloric intake before PN, median (IQR)		30.0 (25.0, 39.0)	35.0 (31.5, 40.3)	31.0 (24.5, 40.3)	0.200
Timeline of PN, median (IQR)	Days from enteral feeding cessation to PN initiation (days)	2.0 (1.0, 3.0)	1.0 (1.0, 3.0)	1.0 (1.0, 2.3)	0.052
	Days from initiating of PN to start feeding	7.0 (6.0, 10.0)	3.5 (1.8, 6.0)	6.0 (4.0, 8.3)	<0.01 **
	Days from PN excluding to full enteral feeding	3.0 (2.0, 5.0)	2.0 (1.0, 3.0)	4.0 (2.0, 8.0)	0.019 *
Interruption of PN, *N* (%)	No	26 (37.1%)	21 (30.0%)	23 (32.9%)	0.037 *
	Yes	13 (61.9%)	1 (4.8%)	7 (33.3%)	
Venous access, *N* (%)	PVC	15 (32.6%)	19 (41.3%)	12 (26.1%)	0.001 **
	CVC	24 (53.3%)	3 (6.7%)	18 (40.0%)	
**Complications and Outcomes**					
CRBSI, *N* (%)	No	20 (30.3%)	21 (31.8%)	25 (37.9%)	<0.01 **
	Yes	19 (76.0%)	1 (4.0%)	5 (20.0%)	
IFALD, median (IQR)	No	31 (40.3%)	18 (23.4%)	28 (36.4%)	0.263
	Yes	8 (57.1%)	4 (28.6%)	2 (14.3%)	
Weight-gain velocity (g/kg/day), median (Q1,Q3)		4.1 (1.4, 6.5)	5.3 (3.9, 7.0)	8.2 (3.1, 11.1)	0.012 *
LOS, median (IQR)		34.0 (29.0)	17.0 (14.5)	17.0 (19.8)	<0.001 **

* *p* < 0.05 ** *p* < 0.01, AAF, Aminoacid-based formulas; CRBSIs, Catheter-related bloodstream infections; CVC, Central vein catheter; eHF, Extensively hydrolyzed protein formula; EN, enteral nutrition; IFALD, Intestinal Failure-Associated Liver Disease; MV, Mechanical ventilation; NEC, necrotizing enterocolitis; NICU, neonatal intensive care; LOS, Length of Stay; PN, Parenteral Nutrition; PVC, Peripheral vein catheter.

## Data Availability

Original data are available on request from the corresponding author.
